# Development of the nervous system in Solenogastres (Mollusca) reveals putative ancestral spiralian features

**DOI:** 10.1186/2041-9139-5-48

**Published:** 2014-12-24

**Authors:** Emanuel Redl, Maik Scherholz, Christiane Todt, Tim Wollesen, Andreas Wanninger

**Affiliations:** Faculty of Life Sciences, Department of Integrative Zoology, University of Vienna, Althanstraße 14, 1090 Vienna, Austria; The Natural History Collections, University of Bergen, University Museum, Allégaten 41, 5007 Bergen, Norway

**Keywords:** Aplacophora, Neomeniomorpha, segmentation, apical organ, evolution, last common spiralian ancestor

## Abstract

**Background:**

The Solenogastres (or Neomeniomorpha) are a taxon of aplacophoran molluscs with contentious phylogenetic placement. Since available developmental data on non-conchiferan (that is, aculiferan) molluscs mainly stem from polyplacophorans, data on aplacophorans are needed to clarify evolutionary questions concerning the morphological features of the last common ancestor (LCA) of the Aculifera and the entire Mollusca. We therefore investigated the development of the nervous system in two solenogasters, *Wirenia argentea* and *Gymnomenia pellucida*, using immunocytochemistry and electron microscopy.

**Results:**

Nervous system formation starts simultaneously from the apical and abapical pole of the larva with the development of a few cells of the apical organ and a posterior neurogenic domain. A pair of neurite bundles grows out from both the neuropil of the apical organ and the posterior neurogenic domain. After their fusion in the region of the prototroch, which is innervated by an underlying serotonin-like immunoreactive (−LIR) plexus, the larva exhibits two longitudinal neurite bundles - the future lateral nerve cords. The apical organ in its fully developed state exhibits approximately 8 to 10 flask-shaped cells but no peripheral cells. The entire ventral nervous system, which includes a pair of longitudinal neurite bundles (the future ventral nerve cords) and a serotonin-LIR ventromedian nerve plexus, appears simultaneously and is established after the lateral nervous system. During metamorphosis the apical organ and the prototrochal nerve plexus are lost.

**Conclusions:**

The development of the nervous system in early solenogaster larvae shows striking similarities to other spiralians, especially polychaetes, in exhibiting an apical organ with flask-shaped cells, a single pair of longitudinal neurite bundles, a serotonin-LIR innervation of the prototroch, and formation of these structures from an anterior and a posterior neurogenic domain. This provides evidence for an ancestral spiralian pattern of early nervous system development and a LCA of the Spiralia with a single pair of nerve cords. In later nervous system development, however, the annelids deviate from all other spiralians including solenogasters in forming a posterior growth zone, which initiates teloblastic growth. Since this mode of organogenesis is confined to annelids, we conclude that the LCA of both molluscs and spiralians was unsegmented.

## Background

The Solenogastres (or Neomeniomorpha) are one of the two aplacophoran, that is, vermiform, sclerite-bearing but shell-less, molluscan taxa with contentious phylogenetic placement (the other being the Caudofoveata (or Chaetodermomorpha); for example, see [1, 2] for a general account of both groups). Some authors have proposed a paraphyletic aplacophoran assemblage at the base of the molluscan tree with either the Solenogastres or the Caudofoveata as the earliest extant offshoot and a monophyletic taxon termed Testaria comprising the remaining representatives, that is, the polyplacophorans and the conchiferans [3–7]. Several recent molecular phylogenetic studies, on the contrary, have shown a basal bifurcation into the Aculifera (comprising the monophyletic Aplacophora and the Polyplacophora) and the Conchifera [8–10] - a view that had already been expressed earlier by some morphologists and that has recently found some support in developmental data [11–14]. Other molecular studies have led to different hypotheses that have received lesser attention [15–17].

Partly as a consequence of this disagreement, the evolutionary emergence of the Mollusca remains unclear. Some authors have proposed that molluscs stem from unsegmented organisms (for example, [3, 18–21]). This is supported by morphological similarities between molluscs and entoprocts, especially between the entoproct creeping-type larva on the one hand and the larva of polyplacophorans, as well as adult solenogasters, on the other. It was thus hypothesized that the (unsegmented) Entoprocta and the Mollusca form a monophyletic taxon termed Lacunifera or Tetraneuralia [21–27]. Others, on the contrary, have argued in favor of a segmented, annelid-like molluscan ancestry, mainly owing to the occurrence of serially repeated organs in some molluscan taxa, in particular in the polyplacophorans and monoplacophorans (for example, [28–32]). This notion is in line with the view of some developmental geneticists that segmentation was a feature of the last common ancestor (LCA) of protostomes or even bilaterians [33–35]. If this is true, loss of segmentation must be a common event in animal evolution, and such cases were indeed reported. For example, molecular phylogenetic studies showed that the Echiura and the Sipuncula, two unsegmented taxa, belong to the Annelida, and developmental data demonstrated that traits of annelid-like segmentation (used here in the sense of a coordinated seriality of several organ systems that develops in an anterior to posterior progression from a posterior growth zone) occur during echiuran and sipunculan nervous system development [36–42]. However, in the Polyplacophora, developmental studies did not reveal any signs of a similar segmental formation of the serially arranged shell plates, muscles, or pedal commissures [43–45]. These findings are corroborated by recent data on myogenesis in *Wirenia argentea*, one of the two solenogaster species analyzed herein, which likewise do not show such a segmental pattern [14].

The studies mentioned above show that ontogeny is a powerful tool to contribute to the clarification of the evolutionary history of a given taxon, but developmental data on the Solenogastres are few, and hardly any are available on nervous system development. The first brief reports on solenogaster development were published some 120 years ago and were followed by more comprehensive studies on different species [46–54]. Accordingly, solenogasters usually develop via a lecithotrophic pericalymma (or test cell) larva, which is characterized by the possession of a calymma (larval test, apical cap) - a larval organ that bears the prototroch and encloses the developing body of the juvenile to a greater or lesser extent. In order to describe the larval nervous system of the Solenogastres and to further test the hypotheses on segmented or unsegmented ancestry of molluscs, we investigated the development of the nervous system in two species of solenogasters, *Wirenia argentea* Odhner, 1921 and *Gymnomenia pellucida* Odhner, 1921 [55].

## Methods

### Immunocytochemistry and confocal laser scanning microscopy

Sediment samples were dredged from the muddy bottom in Hauglandsosen (190-226 m depth) and Hjeltefjorden (227-312 m depth) in the vicinity of Bergen, Norway, using a modified R-P sled [56] with 0.5 or 1 mm net mesh size during January to March 2012 and November 2012 to January 2013. Immediately after collection, the fraction between 5 mm and 350 or 500 μm was isolated by sieving and kept in deep water taken from the sampling location. Specimens of *Wirenia argentea* and *Gymnomenia pellucida* were isolated in the lab and transferred to 50 ml plastic jars. A total of 20 to 35 animals were kept per jar at approximately 4 or 7°C in a fridge in the dark with exposure to light only during handling. 30 to 50% of the water was changed every other day using 20 μm-filtered and UV-sterilized sea water with a salinity of 35‰ (FSSW). The hermaphroditic animals spawned fertilized, uncleaved eggs, allowing the entire development to be traced. Newly hatched larvae were isolated every 12 to 48 h from the cultures, put into separate jars with FSSW and kept under the same conditions as the adults. Voucher specimens of adult animals of both species from an earlier collection at the locality in Hauglandsosen are deposited in the Natural History Collections of the University Museum of Bergen, Norway (Collection numbers ZMBN 94730 for *W. argentea* and ZMBN 94742–94744 for *G. pellucida*). Barcoding data from these specimens are available in the Barcode of Life Data System (BOLD) [57] (BOLD IDs UM_NB_aplac76 for *W. argentea* and UM_NB_aplac88-90 for *G. pellucida*).

Larvae were fixed with 4% paraformaldehyde in 0.1 M phosphate buffer (pH = 7.3) for 1 to 3 h at room temperature (RT) or 4°C. Specimens from an age of 8 days posthatching (dph) onwards were relaxed prior to fixation for 20 min to 1 h at 4°C by adding a 3.2% magnesium chloride solution. The samples were subsequently rinsed three times at RT or 4°C in 0.1 M phosphate buffer (pH = 7.3) with 0.1% sodium azide for a total period of 30 min to 1 h (or overnight at 4°C) and stored in 0.1 M phosphate buffer with 0.1% sodium azide (pH = 7.3) at 4°C. Adult specimens were relaxed for 30 min, fixed for 2 h, rinsed two times for a total period of 10 to 30 min (all steps at 4°C) and otherwise treated as the larvae.

For immunocytochemical labeling, larvae with an age of 3 dph or older were decalcified in 0.05 M EGTA (pH = 7.3) for 1 to 2 h at RT. All larvae were rinsed and permeabilized in 0.1 M phosphate buffer (pH = 7.3) with 4% Triton X-100 and 0.1% sodium azide (PTA) at RT for 45 min to 2 h (with two changes of the permeabilization solution in case of previous decalcification). Blocking of unspecific binding sites was done with a 6% solution of normal goat serum (Jackson ImmunoResearch, West Grove, PA, USA, or Invitrogen - Life Technologies, Carlsbad, CA, USA) in PTA (block-PTA) for 12 to 24 h at RT. Primary antibodies used were rabbit anti-serotonin (5-HT; polyclonal; Sigma-Aldrich, St. Louis, MO, USA, or ImmunoStar, Hudson, WI, USA), rabbit anti-FMRF-amide (polyclonal; ImmunoStar, or Biotrend, Cologne, Germany), and mouse anti-acetylated α-tubulin (monoclonal; Sigma-Aldrich), whereby the last-mentioned antibody labels not only neural but also ciliary structures (see, for example, [44, 54, 58]). After blocking of the samples, primary antibodies were applied in a dilution of 1:400 to 1:800 (anti-serotonin) or 1:400 (anti-FMRF-amide, anti-acetylated α-tubulin) in block-PTA for 23 to 30 h at RT. Hereby, most larvae were double-labeled with a mixture of two antibodies, that is, with either rabbit anti-serotonin or anti-FMRF-amide in combination with mouse anti-acetylated α-tubulin. The larvae were subsequently rinsed four times at RT in block-PTA for a total period of 5 to 29 h before applying the secondary antibodies, which included Alexa Fluor 488, 568 and 633 goat anti-rabbit as well as anti-mouse antibodies (all from Molecular Probes - Life Technologies, Carlsbad, CA, USA). All secondary antibodies were applied in a 1:200 dilution in block-PTA and the larvae were incubated for 24 to 42 h at RT (this and all subsequent steps were done in the dark). In accordance with the primary antibody treatment, most larvae were double-labeled, that is, treated with a mixture of one anti-rabbit and one anti-mouse antibody labeled with different fluorescent dyes. The larvae were then rinsed four times at RT or 4°C in 0.1 M phosphate buffer (pH = 7.3) for a total period of 4 to 27 h. Some specimens were additionally stained with DAPI (Sigma-Aldrich, or Molecular Probes - Life Technologies) in a final concentration of 1 to 60 μg/ml, which was added either to the secondary antibody solution or to the phosphate buffer in the first step of the final washing procedure. The larvae were mounted in Fluoromount-G (SouthernBiotech, Birmingham, AL, USA) on microscope slides and were stored until examination at 4°C in the dark. Adult specimens were treated identically to the larvae.

Negative controls were performed by omitting either the primary or both antibodies. No signal was detected in any of these preparations. In order to test for unspecific binding of the anti-serotonin (5-HT) antibodies, additional negative controls with preadsorbed antibodies were performed on developmental stages of *W. argentea* exhibiting a ventromedian nerve plexus. For this, the rabbit anti-serotonin (5-HT) antibody (polyclonal; ImmunoStar) was incubated in block-PTA for 23 h at 4°C together with serotonin- (5-HT-)BSA conjugate (ImmunoStar) reconstituted in block-PTA with a final dilution of the antibody of 1:500 and a final concentration of the serotonin-BSA conjugate of 20 μg/ml. This solution was subsequently used as primary antibody solution according to the protocol described above and none of the animals showed any signal.

The analysis of all preparations was performed on a Leica TCS SP5 II confocal laser scanning microscope equipped with the software Leica Application Suite Advanced Fluorescence (LAS AF), Version 2.6.0 (Leica Microsystems, Wetzlar, Germany). Approximately 300 specimens were investigated in total. The obtained image data were further processed with the LAS AF software as well as with Adobe Photoshop CS5 Extended, Version 12.0 x64 (Adobe Systems, San José, CA, USA). The schematic drawings were generated with Adobe Illustrator CS5, Version 15.0.0 (Adobe Systems).

### Transmission electron microscopy

From September to November 2007, adult specimens of *Wirenia argentea* were collected and subsequently cultured as described in [54]. The larvae were relaxed for 20 to 25 min by drop-wise addition of cold 7.14% magnesium chloride solution and fixed for 12 h at 7°C in 4% glutardialdehyde in 0.2 M sodium cacodylate buffer (pH = 7.3) with 0.1 mol/l NaCl and 0.35 mol/l sucrose added. They were rinsed three times for 10 min at 7°C in this buffer diluted 1:1 with distilled water, postfixed for 1.5 h on ice in 1% osmium tetroxide in filtered sea water, rinsed again three times for 5 to 10 min in distilled water at RT and subsequently dehydrated in a graded ethanol series with 50%, 70%, 80%, 90% and 100% ethanol steps (each step three times for 5 min at RT; the larvae were stored in 70% ethanol at RT). The larvae were then infiltrated at RT with 100% propylene oxide (three times for 5 min), followed by mixtures of propylene oxide and agar low viscosity resin (Agar Scientific, Stansted, Essex, UK; mixture for medium hardness) in a ratio of 2:1 for 2 to 3 hours and in a ratio of 1:1 overnight (with an open lid to let the propylene oxide evaporate). The next day, the infiltration was continued in pure resin for 6 h, and the larvae were subsequently transferred to an embedding mold with fresh resin, which was polymerized for 18 h at 60°C.

Ultrathin sections with a thickness of 60 to 120 nm were cut on a Reichert-Jung Ultracut microtome with a Diatome Ultra 45° diamond knife (Diatome, Biel, Switzerland). They were mounted on formvar-coated mesh grids (sometimes additionally coated with carbon), contrasted with 1% or 2% uranyl acetate for 40 to 60 min, rinsed thoroughly with distilled water, air dried and contrasted again with lead citrate (after [59]) for 8 to 16 min. The sections were analyzed and documented on a JEM-1011 transmission electron microscope (JEOL, Akishima, Tokyo, Japan) equipped with a Morada Soft Imaging System (Olympus Corporation, Shinjuku, Tokyo, Japan). Further image processing was done with Adobe Photoshop CS5 Extended, Version 12.0 x64 (Adobe Systems).

## Results

### General remarks

Larval morphology and timing of development followed a highly similar pattern in both species investigated (cf. [54]). Differences in nervous system development concerned only a few minor aspects, and these are specifically mentioned where they occurred.

### Acetylated α-tubulin-like immunoreactive nervous system

**Figure 1:**
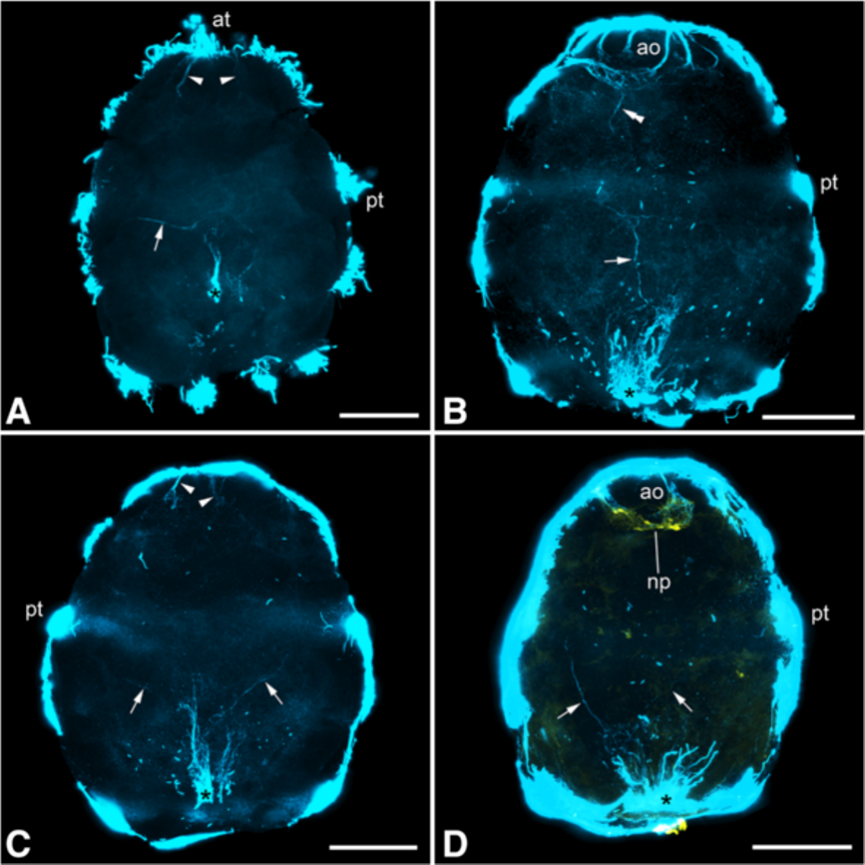
**Immunolabeling of larvae of** ***Wirenia argentea.*** Maximum intensity projections of confocal image stacks; apical is up and scale bar equals 40 μm in all panels. **A**: Labeling of acetylated α-tubulin-like immunoreactive (−LIR) components; 0 to 1 days posthatching (dph) larva showing developing apical organ (arrowheads) and abapical neurogenic domain (asterisk) with outgrowing neurite bundle (arrow). **B**: Labeling of acetylated α-tubulin-LIR components; 1 to 2 dph larva showing abapical neurogenic domain (asterisk) with outgrowing neurite bundle (arrow) as well as a second neurite bundle (double arrowhead) growing out from the neuropil of the apical organ. **C**: Labeling of acetylated α-tubulin-LIR components; 1 to 2 dph larva showing developing apical organ (arrowheads) and abapical neurogenic domain (asterisk) with a pair of outgrowing neurite bundles (arrows). **D**: Labeling of acetylated α-tubulin-LIR (blue) and FMRF-amide-LIR (yellow) components; 2 to 3 dph larva showing abapical neurogenic domain (asterisk) with a pair of outgrowing neurite bundles (arrows). Abbreviations: ao, apical organ; at, apical tuft; np, neuropil of apical organ; pt, prototroch.

**Figure 2:**
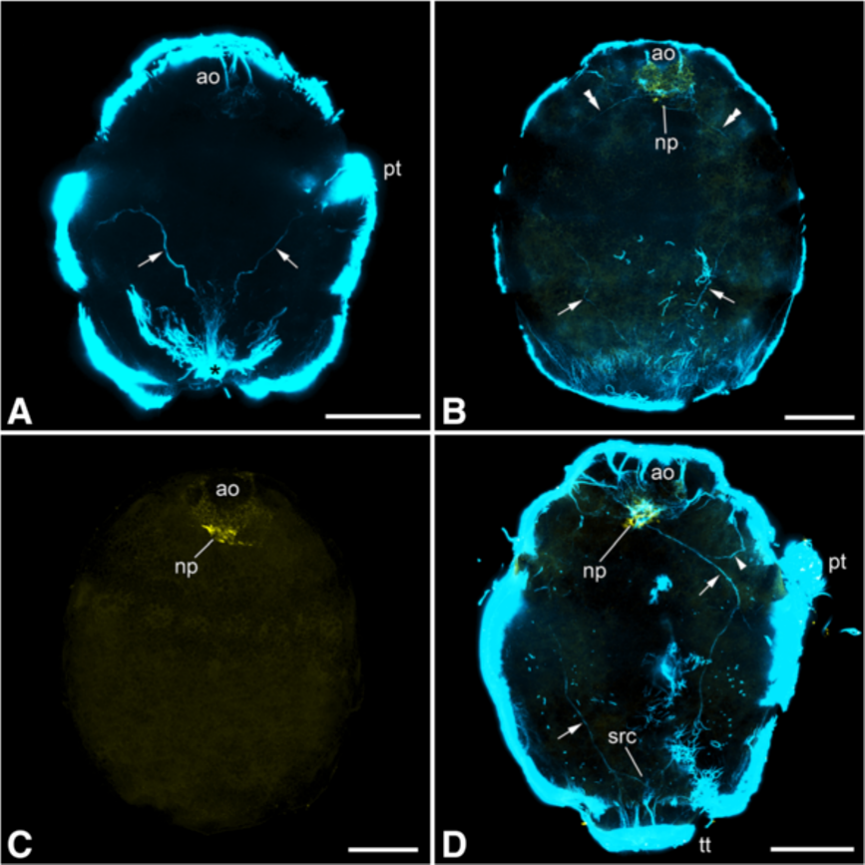
**Immunolabeling of larvae of** ***Gymnomenia pellucida.*** Maximum intensity projections of confocal image stacks; apical is up and scale bar equals 40 μm in all panels. **A**: Labeling of acetylated α-tubulin-like immunoreactive (−LIR) components; 1 to 2 days posthatching (dph) larva showing abapical neurogenic domain (asterisk) with an outgrowing pair of neurite bundles (arrows). **B**: Labeling of acetylated α-tubulin-LIR (blue) and FMRF-amide-LIR (yellow) components; 2 to 3 dph larva showing a pair of abapically outgrowing neurite bundles (arrows) as well as a second pair of neurite bundles (double arrowheads) growing out from the neuropil of the apical organ. **C**: Labeling of FMRF-amide-LIR components; 2 to 3 dph larva. **D**: Labeling of acetylated α-tubulin-LIR (blue) and FMRF-amide-LIR (yellow) components; 6 to 7 dph larva scanned in ventral aspect showing lateral neurite bundles (arrows) with neurite (arrowhead) branching off to the lateral part of the apical region; note that the neurite bundle on the right side of the animal is not yet fully developed. Abbreviations: ao, apical organ; np, neuropil of apical organ; pt, prototroch; src, suprarectal commissure; tt, telotroch.

**Figure 3:**
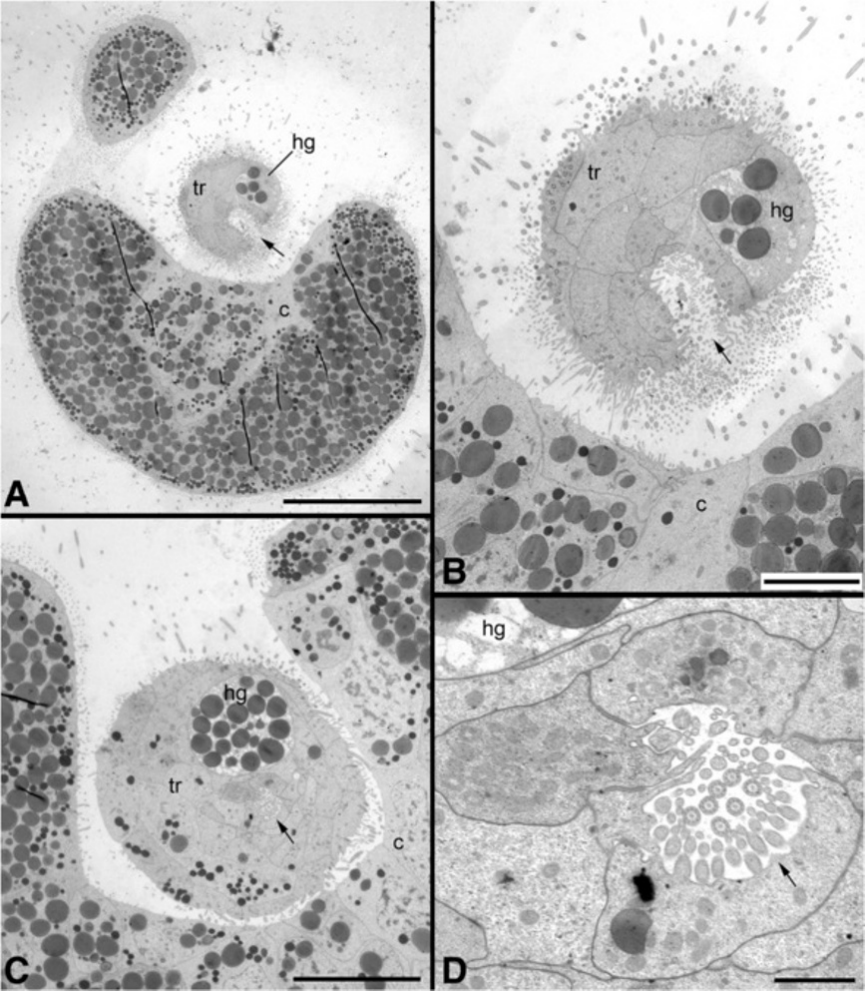
**Transmission electron micrographs of ultrathin sections of a 0 to 3 days posthatching larva of** ***Wirenia argentea*** **. A**: Cross section through the abapical part showing the opening of the posterior invagination (arrow) to the outside; scale bar equals 20 μm. **B**: Detail from A showing the opening of the posterior invagination (arrow) to the outside; scale bar equals 5 μm. **C**: Part of a cross section through the abapical part somewhat more apical than in A showing the posterior invagination (arrow); scale bar equals 10 μm. **D**: Detail from C showing the posterior invagination (arrow); scale bar equals 1 μm. Abbreviations: c, calymma; hg, hindgut anlage; tr, trunk.

**Figure 4:**
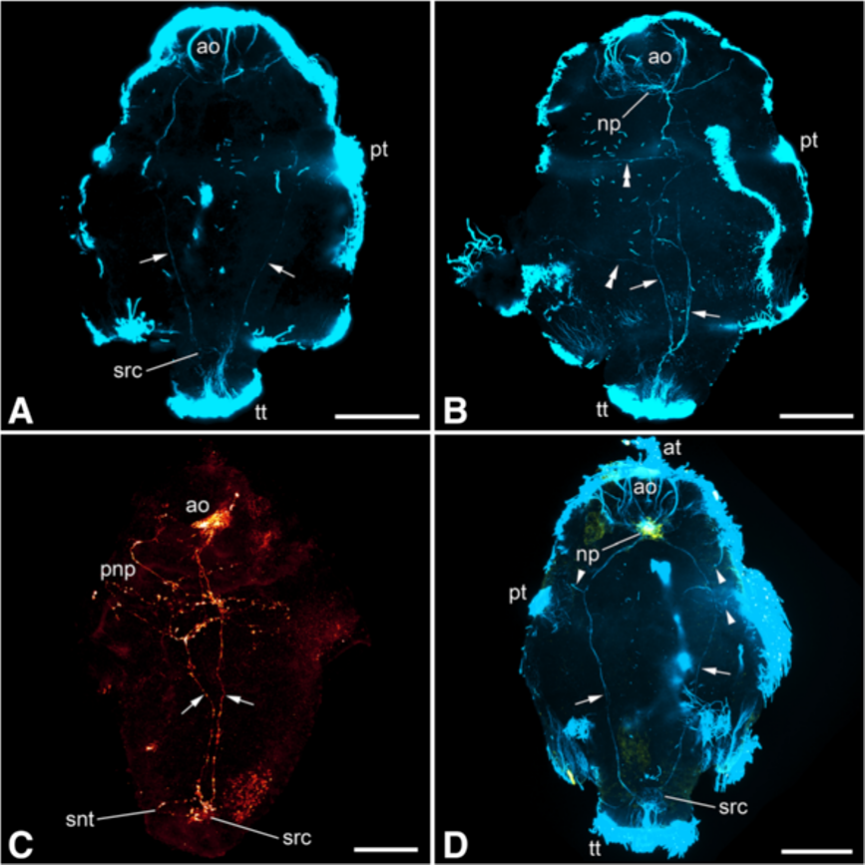
**Immunolabeling of larvae of** ***Wirenia argentea*** **.** Maximum intensity projections of confocal image stacks; apical is up and scale bar equals 40 μm in all panels. **A**: Labeling of acetylated α-tubulin-like immunoreactive (−LIR) components; 4 to 5 days posthatching (dph) larva scanned in ventral aspect showing lateral neurite bundles (arrows); note that the neurite bundle on the left side of the animal is not yet fully developed. **B**: Labeling of acetylated α-tubulin-LIR components; 6 to 7 dph larva scanned in right lateral aspect showing lateral neurite bundles (arrows) with neurites (double arrowheads) branching off to the dorsal parts of the animal. **C**: Labeling of serotonin-LIR components; 11 to 12 dph larva scanned in left lateral aspect showing signal in the apical organ, the prototrochal nerve plexus, the lateral neurite bundles (arrows), and the suprarectal commissure. **D**: Labeling of acetylated α-tubulin-LIR (blue) and FMRF-amide-LIR (yellow) components; 9 to 10 dph larva scanned in ventral aspect showing lateral neurite bundles (arrows) with neurites (arrowheads) branching off to the prototroch and the lateral parts of the pre-trochal region. Abbreviations: ao, apical organ; at, apical tuft; np, neuropil of apical organ; pnp, prototrochal nerve plexus; pt, prototroch; snt, serotonin-LIR neurites to terminal body region; src, suprarectal commissure; tt, telotroch.

**Figure 5:**
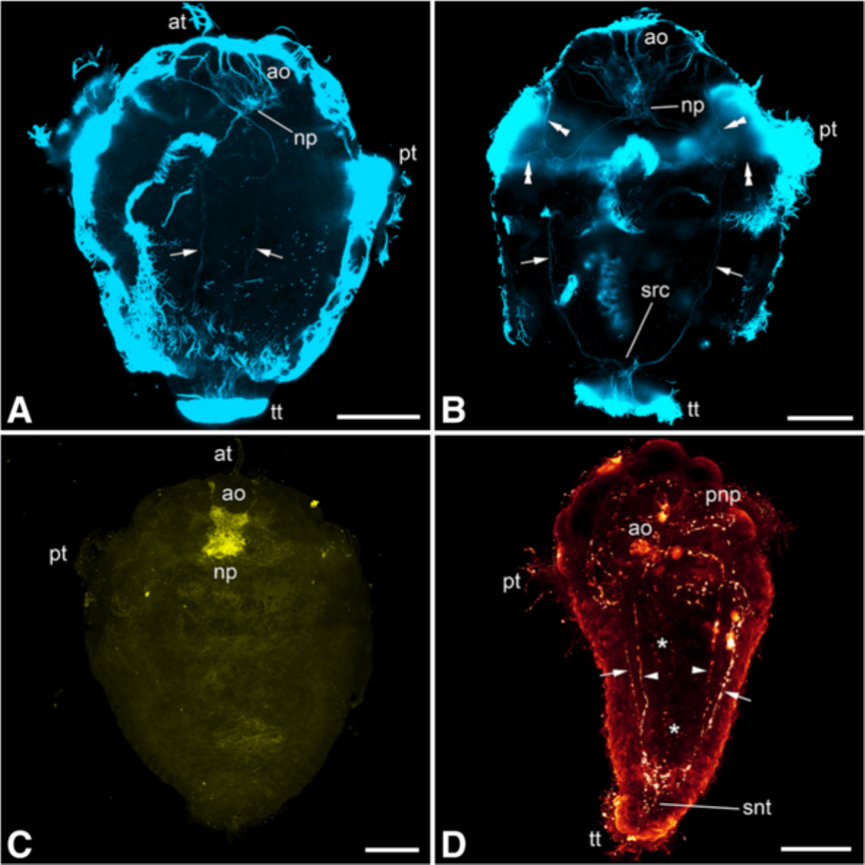
**Immunolabeling of larvae of** ***Gymnomenia pellucida.*** Maximum intensity projections of confocal image stacks; apical is up and scale bar equals 40 μm in all panels. **A**: Labeling of acetylated α-tubulin-like immunoreactive (−LIR) components; 6 to 7 days posthatching (dph) larva scanned in left lateral aspect showing lateral neurite bundles (arrows). **B**: Labeling of acetylated α-tubulin-LIR components; 8 to 9 dph larva scanned in ventral aspect showing lateral neurite bundles (arrows) with neurites (double arrowheads) branching off to the prototroch and the lateral parts of the pre-trochal region. **C**: Labeling of FMRF-amide-LIR components; 9 to 10 dph larva. **D**: Labeling of serotonin-LIR components; 10 to 11 dph larva scanned in dorsal aspect showing lateral (arrows) and ventral (arrowheads) neurite bundles and the ventromedian nerve plexus (asterisks). Abbreviations: ao, apical organ; at, apical tuft; np, neuropil of apical organ; pnp, prototrochal nerve plexus; pt, prototroch; snt, serotonin-LIR neurites to terminal body region; src, suprarectal commissure; tt, telotroch.

**Figure 6:**
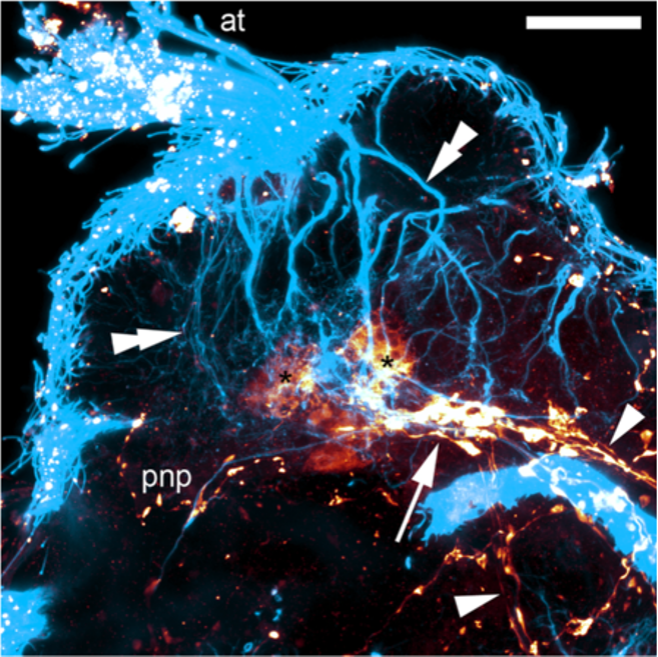
**Immunolabeling of a larva of** ***Wirenia argentea.*** Maximum intensity projection of confocal image stack; apical part upwards; scale bar equals 20 μm. Labeling of acetylated α-tubulin-like immunoreactive (−LIR) (blue) and serotonin-LIR (orange) components; 15 to 16 days posthatching larva, detail of apical organ region scanned in right lateral aspect showing the cerebral commissure (arrow) with the originating longitudinal neurite bundles (arrowheads) and the flask-shaped cells of the apical organ with their projections (double arrowheads) to the surface; note that two of the apical organ cells are serotonin-LIR (asterisks). Abbreviations: at, apical tuft; pnp, prototrochal nerve plexus.

In both species the first structures related to the nervous system are two formation domains that are present in newly hatched larvae and are situated at the apical (anterior) and abapical (posterior) pole, respectively (Figures 1 and 2A-B). The former is represented by the first few flask-shaped cells of the apical organ and its (developing) neuropil. The abapical neurogenic domain yields a strong acetylated α-tubulin-like immunoreactive (-LIR) signal, which may at least in part stem from a terminal invagination bordered by ciliated cells next to the anlage of the hindgut (see Figure 3), and from this region, a pair of neurite bundles grows in an anterior direction (Figures 1 and 2A-B). Subsequently, another pair of neurite bundles starts to grow from the neuropil of the apical organ in a posterior direction, so that the two pairs grow toward each other (Figures 1B and 2B). In slightly further developed specimens, the pre- and post-trochal neurite bundles meet in the area of the prototroch to form a pair of continuous, longitudinal, lateral neurite bundles (Figures 2D, 4 and 5A-B). From the site of fusion, neurites branch off and project towards the prototroch and the lateral parts of the pre-trochal region (Figures 2D, 4D and 5B). The ciliated terminal invagination has disappeared and the two longitudinal neurite bundles are interconnected posteriorly via a neural structure (the future suprarectal commissure) that includes large cells and innervates the terminal body region (Figures 2D, 4A and D and 5B). The apical organ is now fully developed and consists of approximately 8 to 10 large, flask-shaped cells and a distinct basal neuropil (Figures 2D, 4B and D, 5A-B and 6).

**Figure 7:**
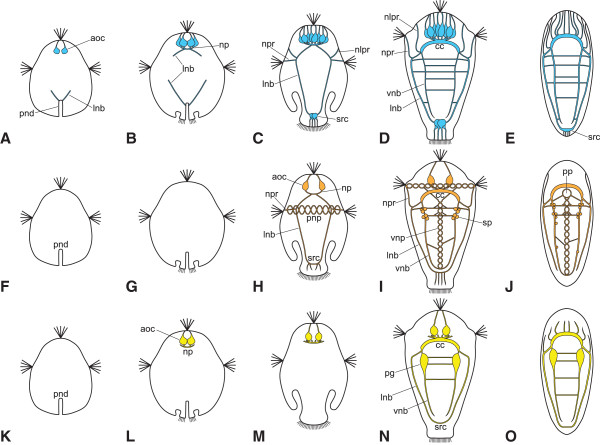
**Summary of nervous system development in** ***Wirenia argentea*** **and** ***Gymnomenia pellucida*** **.** Schematic drawings based on labeling of acetylated α-tubulin-like immunoreactive (−LIR) (A-E), serotonin-LIR (F-J), and FMRF-amide-LIR (K-O) components of the nervous system during the development of the two solenogaster species investigated; dorsal aspect, apical/anterior is up in all images. In stages where neurotransmitter labeling was inconsistent (H, L, M, N for *W. argentea* and I, L, M, N for *G. pellucida*) the maximum amount of labeled structures is depicted; note that serotonin-LIR signal in stage 3 (H) is only present in *W. argentea* but not in *G. pellucida*. The ventromedian as well as the prototrochal nerve plexus are not visible in A-E, probably due to their position right beneath the cilia of the developing foot or the prototroch, respectively, and the resulting signal interference; note that even in the early juvenile stage no part of the buccal nervous system was found (we did, however, find a strong serotonin- and a weak acetylated α-tubulin-LIR signal in the buccal nervous system of some adult specimens; see Figure 11). Abbreviations: aoc, flask-shaped cell of apical organ; cc, cerebral commissure; lnb, lateral neurite bundle(s); nlpr, neurite to lateral parts of pre-trochal region; np, neuropil of apical organ; npr, neurite to prototroch; pg, pedal ganglion; pnd, posterior neurogenic domain; pnp, prototrochal nerve plexus; pp, pedal pit; sp, serotonin-LIR perikaryon; src, suprarectal commissure; vnb, ventral neurite bundle; vnp, ventromedian nerve plexus.

During subsequent development, the cerebral commissure is formed at the base of the apical organ, to which it is connected in its anterior region (Figure 7D). In addition, a second pair of longitudinal neurite bundles appears, and this pair lies more ventrally than the first one. This ventral nervous system is formed *de novo* and appears simultaneously in its entirety without any recognizable intermediate stages. The lateral pair of neurite bundles now appears distinctly larger in diameter than before and is generally more prominent than the ventral pair. The four neurite bundles originate at the cerebral commissure and are interconnected by lateroventral connectives and ventral commissures; up to five ventral commissures were identified. From the lateral sides of the cerebral commissure and the cerebrolateral connectives, neurites project towards the prototroch and the lateral parts of the pre-trochal region (Figure 7D).During metamorphosis, the flask-shaped cells of the apical organ and the large cells associated with the suprarectal commissure are lost. In early juveniles, several neurites project from the cerebral and the suprarectal commissure into the anterior or terminal body region, respectively (Figure 7E). The lateral and ventral neurite bundles are now of more or less equal thickness.

### Serotonin-like immunoreactive nervous system

**Figure 8:**
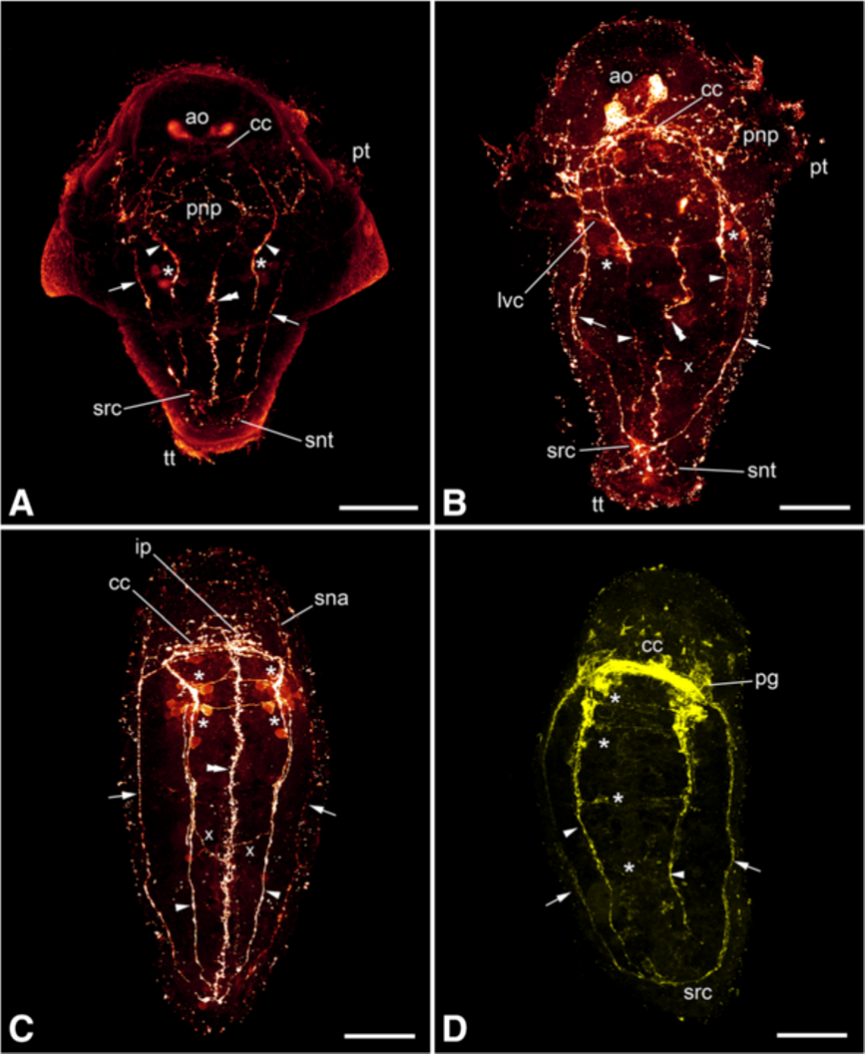
**Immunolabeling of larvae and early juveniles of** ***Wirenia argentea.*** Maximum intensity projections of confocal image stacks; apical is up and scale bar equals 40 μm in all panels. **A**: Labeling of serotonin-like immunoreactive (−LIR) components; 15 to 16 days posthatching (dph) larva scanned in ventral aspect showing lateral (arrows) and ventral (arrowheads) neurite bundles and the ventromedian nerve plexus (double arrowhead); note the clusters of perikarya (asterisks) associated with the ventral neurite bundles. **B**: Labeling of serotonin-LIR components; 15 to 16 dph larva scanned in dorsal aspect showing lateral (arrows) and ventral (arrowheads) neurite bundles and the ventromedian nerve plexus (double arrowhead); note the ventral commissures associated with the clusters of perikarya (asterisks) on the ventral neurite bundles and the additional connections between the ventral neurite bundles and the ventromedian nerve plexus (x). **C**: Labeling of serotonin-LIR components; 19 to 21 dph early juvenile stage scanned in ventral aspect showing lateral (arrows) and ventral (arrowheads) neurite bundles and the ventromedian nerve plexus (double arrowhead); note the ventral commissures associated with clusters of perikarya (asterisks) at the ventral neurite bundles and the additional connections between the ventral neurite bundles and the ventromedian nerve plexus (x). **D**: Labeling of FMRF-amide-LIR components; 19 to 21 dph early juvenile stage scanned in ventral aspect showing lateral (arrows) and ventral (arrowheads) neurite bundles; note the ventral commissures (asterisks) and the fact that the ventromedian nerve plexus is not labeled. Abbreviations: ao, apical organ; cc, cerebral commissure; ip, innervation of pedal pit; lvc, lateroventral connective; pg, pedal ganglion; pnp, prototrochal nerve plexus; pt, prototroch; sna, serotonin-LIR neurites to anterior body region; snt, serotonin-LIR neurites to terminal body region; src, suprarectal commissure; tt, telotroch.

**Figure 9:**
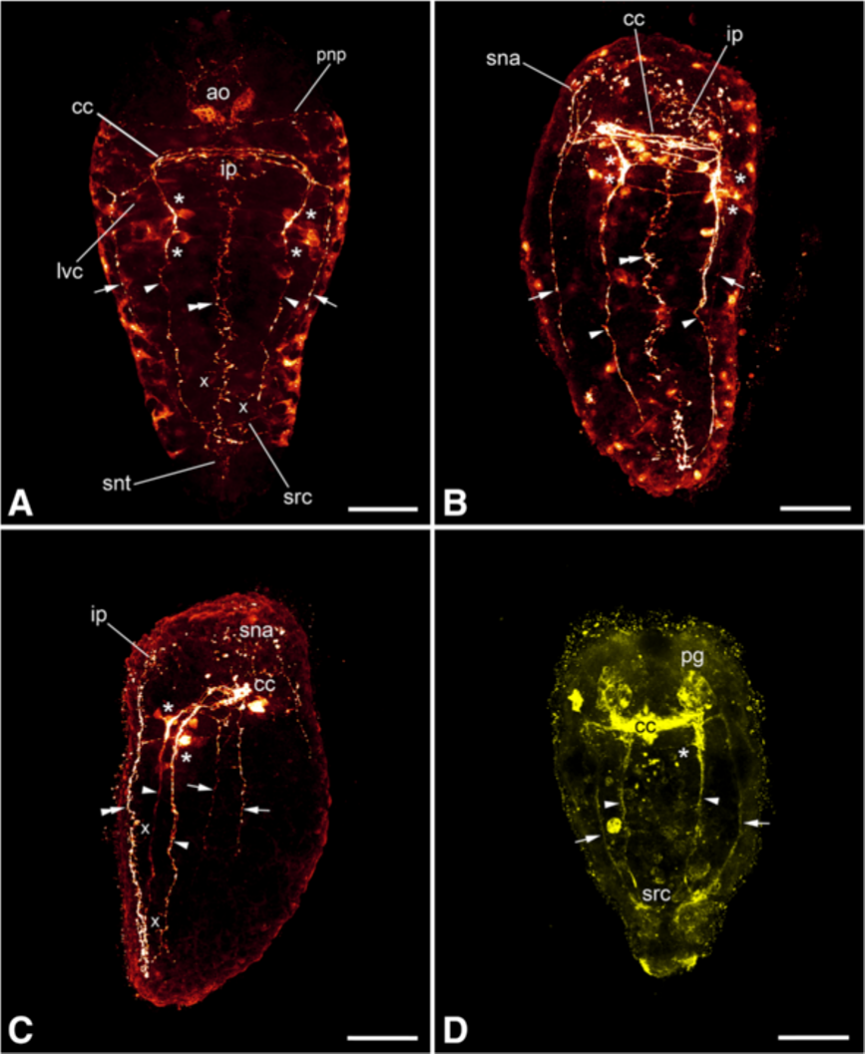
**Immunolabeling of a late larva and early juveniles of** ***Gymnomenia pellucida.*** Maximum intensity projections of confocal image stacks; apical is up and scale bar equals 40 μm in all panels. **A**: Labeling of serotonin-like immunoreactive (−LIR) components; 15 to 16 days posthatching (dph) larva scanned in ventral aspect showing lateral (arrows) and ventral (arrowheads) neurite bundles and the ventromedian nerve plexus (double arrowhead); note the ventral commissures associated with clusters of perikarya (asterisks) on the ventral neurite bundles and the additional connections between the ventral neurite bundles and the ventromedian nerve plexus (x). **B**: Labeling of serotonin-LIR components; 19 to 20 dph early juvenile stage scanned in ventral aspect showing lateral (arrows) and ventral (arrowheads) neurite bundles and the ventromedian nerve plexus (double arrowhead); note the ventral commissures associated with clusters of perikarya (asterisks) on the ventral neurite bundles. **C**: Labeling of serotonin-LIR components; 19 to 20 dph early juvenile stage scanned in left lateral aspect showing lateral (arrows) and ventral (arrowheads) neurite bundles and the ventromedian nerve plexus (double arrowhead); note the ventral commissures associated with clusters of perikarya (asterisks) on the ventral neurite bundles and the additional connections between the ventral neurite bundles and the ventromedian nerve plexus (x). **D**: Labeling of FMRF-amide-LIR components; 19 to 20 dph early juvenile stage scanned in ventral aspect showing lateral (arrows) and ventral (arrowheads) neurite bundles; note the ventral commissure (asterisk) and the fact that the ventromedian nerve plexus is not labeled. Abbreviations: ao, apical organ; cc, cerebral commissure; ip, innervation of pedal pit; lvc, lateroventral connective; pg, pedal ganglion; pnp, prototrochal nerve plexus; sna, serotonin-LIR neurites to anterior body region; snt, serotonin-LIR neurites to terminal body region; src, suprarectal commissure.

**Figure 10:**
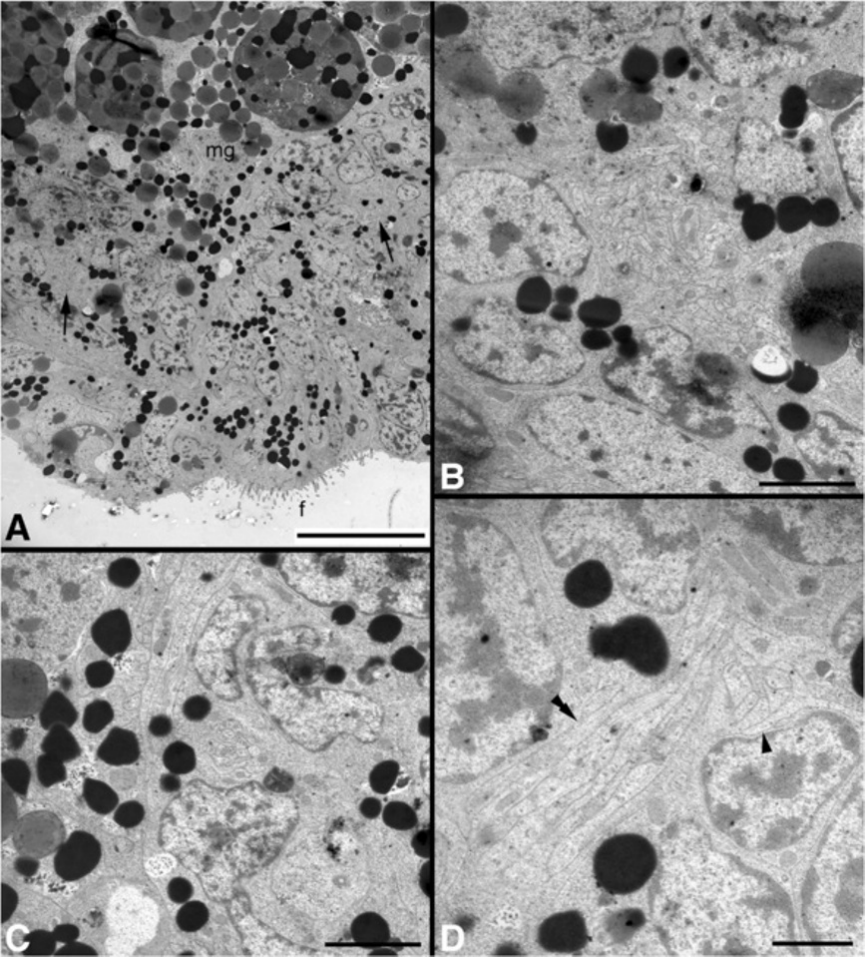
**Transmission electron micrographs of ultrathin sections of a 10 to 16 days posthatching larva of** ***Wirenia argentea*** **. A**: Part of a cross section through the middle part showing the pair of ventral neurite bundles (arrows) and a ventromedian neurite bundle (arrowhead) belonging to the ventromedian nerve plexus; scale bar equals 10 μm. **B**: Detail from A showing one of the ventral neurite bundles; scale bar equals 2 μm. **C**: Detail from A showing the ventromedian neurite bundle belonging to the ventromedian nerve plexus; scale bar equals 2 μm. **D**: Detail from a cross section through the middle part somewhat more posterior than in A, showing two neurite bundles of the ventromedian nerve plexus, one in cross section (arrowhead) and one in oblique section (double arrowhead), emphasizing the plexus-like character of this fifth concentration of nervous material (compare also Figures 5D, 8A-C and 9A-C); scale bar equals 1 μm. Abbreviations: f, foot; mg, midgut anlage.

No serotonin-LIR signal was found in early stages, that is, prior to the establishment of the first pair of longitudinal neurite bundles, in either of the two species investigated. Only in specimens where the lateral neurite bundles have formed completely, the main parts of the nervous system, that is, the neurite bundles, the neuropil and two flask-shaped cells of the apical organ, and the suprarectal commissure are sometimes labeled in *Wirenia argentea*. In addition, there is a prototrochal nerve plexus that originates from the fusion point of the pre- and post-trochal parts of the nervous system and underlies the prototroch (Figure 4C). In the suprarectal commissure only neurites but no perikarya are visible, with a few of them branching off and innervating the terminal body region. The neuropil at the base of the apical organ is only partially labeled and shows neurites that interconnect the serotonin-LIR cells of the apical organ. In *Gymnomenia pellucida*, in contrast, unambiguous identification of definite serotonin-LIR nervous structures was not possible prior to the establishment of the ventral parts of the nervous system. Generally, labeling of the larvae showed a high individual variation even among specimens that belonged to the same age group and had been treated identically during the entire process from relaxation to immunocytochemical labeling. A consistent signal was observed only after the formation of the ventral nervous system in *W. argentea* or after metamorphosis in *G. pellucida*.After the development of the ventral nervous system, the ventral pair of neurite bundles and the cerebral commissure show a distinct signal in addition to the above-mentioned structures. Furthermore, a narrow, longitudinal nerve plexus appears ventromedially, immediately above the developing foot (Figures 5D, 6, 8A-C and 9A-C). This developing plexus was also found in TEM sections as a small bundle of neurites that lies ventrally to the gut anlage (Figure 10). As with acetylated α-tubulin-LIR labeling, the serotonin-LIR components of the ventral nervous system appear simultaneously. Two ventral commissures with associated clusters of perikarya and connections to the ventromedian nerve plexus and a pair of lateroventral connectives are visible in the anterior part of the longitudinal neurite bundles (Figures 8A-C and 9A-C). In addition, there are independent neural connections between the ventral neurite bundles and the ventromedian nerve plexus (Figures 8B and C and 9A and C). The anterior parts of the ventromedian nerve plexus are connected to the prototrochal nerve plexus (Figure 7I).

**Figure 11:**
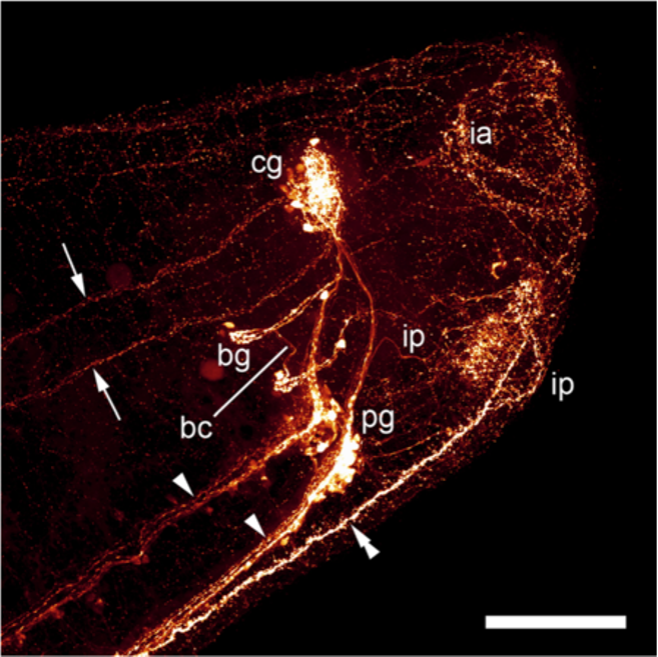
**Immunolabeling of an adult specimen of** ***Wirenia argentea.*** Maximum intensity projection of confocal image stack; anterior to the right; scale bar equals 100 μm. Labeling of serotonin-like immunoreactive components, anterior body region scanned in right lateral aspect showing lateral (arrows) and ventral (arrowheads) nerve cords and the ventromedian nerve plexus (double arrowhead). Abbreviations: bc, buccal connective; bg, buccal ganglion; cg, cerebral ganglion; ia, innervation of atrial sense organ; ip, innervation of pedal pit; pg, pedal ganglion.

After metamorphosis, the flask-shaped cells of the apical organ and the prototrochal nerve plexus have disappeared (Figures 8C and 9B-C). Serotonin-LIR neurites lead from the former point of origin of the prototrochal nerve plexus to the anterior body region (Figures 8C and 9B-C). The ventromedian nerve plexus extends anteriorly and innervates the pedal pit together with nerves from the cerebroventral connectives (Figures 8C, 9B-C and 11). Single serotonin-LIR perikarya are sometimes present along the ventral neurite bundles in addition to the above-mentioned clusters (Figure 7J). The signal of the ventral nervous system is generally more prominent than that of the lateral one (Figures 8C and 9B-C). The suprarectal commissure and its associated neurites have ceased to express serotonin-like immunoreactivity.

### FMRF-amide-like immunoreactive nervous system

The first signal appears in the apical organ, starting from an age of about 2 to 3 days posthatching (dph). In some larvae, two faintly labeled, FMRF-amide-LIR flask-shaped cells are visible, but the associated neuropil is labeled more consistently and generally yields a much stronger (and sometimes, the only) signal. This situation persists until the first pair of longitudinal neurite bundles has formed (Figures 1D, 2B-D, 4D and 5C). As development proceeds and metamorphosis approaches, the signal variably extends to the cerebral commissure and the four longitudinal neurite bundles, including the neurites of the suprarectal commissure, a pair of large pedal ganglia at the anterior end of the ventral neurite bundles, and usually three ventral commissures, but never to the ventromedian and the prototrochal nerve plexus (Figure 7N). Labeling of the larvae showed a high individual variation even among specimens that belonged to the same age group and had been treated identically during the entire process from relaxation to immunocytochemical labeling. A consistent signal was observed only after metamorphosis. At this stage, the flask-shaped cells of the apical organ have disappeared and a few weakly FMRF-amide-LIR neurites run from the cerebral commissure and the cerebrolateral connectives in anterior direction; up to four ventral commissures can be observed (Figures 7O, 8D and 9D).

## Discussion

### Development of the solenogaster nervous system

We have subdivided the nervous system development of solenogasters in five stages (see Figure 7). They correlate approximately with the general morphology of the larvae but correspond only roughly to their absolute age, since the speed of development shows a high individual variation even in batches of larvae reared in the same jar and thus under identical conditions. Stage 1 (Figure 7A, F and K) is characterized by the establishment of the first flask-shaped cells of the apical organ and the posterior neurogenic domain, which includes a terminal invagination bordered by ciliated cells. In addition, the post-trochal portions of the lateral neurite bundles begin to grow out from this region in an anterior direction. In stage 2 (Figure 7B, G and L), the apical organ shows an increase in the number of flask-shaped cells and the formation of a neuropil at the base, from where the pre-trochal portions of the lateral neurite bundles start to grow out in a posterior direction. The fusion of the pre- and post-trochal portions of the lateral neurite bundles in the area of the prototroch and the development of the prototrochal nerve plexus (or at least of its serotonin-LIR components) are the distinguishing features of stage 3 (Figure 7C, H and M). The apical organ has now reached its fully developed state and exhibits 8 to 10 flask-shaped cells. The ciliated terminal invagination has disappeared and the lateral neurite bundles are connected via the suprarectal commissure. In stage 4 (Figures 7D, I and N) the ventral nervous system, which consists of one pair of neurite bundles and a ventromedian, unpaired nerve plexus, and the cerebral commissure at the base of the apical organ have formed. After metamorphosis, the animals reach the early juvenile stage (stage 5; Figures 7E, J and O), which differs from the preceding stage mainly by the loss of the prototrochal nerve plexus and the flask-shaped cells of the apical organ.

A comparison with the description of the adult nervous system of *Wirenia argentea* [60] shows that the main adult neural structures already develop during larval life and that the only elements of the larval nervous system that do not persist through metamorphosis are the prototrochal nerve plexus and the apical organ. The lateral and ventral neurite bundles develop into the lateral and ventral nerve cords, respectively, the neuropil beneath the apical organ develops into (at least part of) the cerebral commissure, and the cellular posterior connection of the lateral neurite bundles becomes the (cord-like) suprarectal commissure. The ventromedian serotonin-LIR nerve plexus was also found in adult specimens of three species of solenogasters including *W. argentea* [60, 61] but was interpreted as a non-neural signal in the former study. Our transmission electron microscopy analysis, however, demonstrates that it constitutes a part of the nervous system, namely a narrow plexus (which superficially resembles a nerve cord) associated with the (developing) creeping sole. Interestingly, a serotonin-LIR ventral plexus was also described in polyplacophorans [43, 44], opisthobranchs [62], pulmonates [63–65], and some polychaete annelids, where it is also associated with a ventral locomotory ciliary band [66]. The neurites projecting from the cerebral commissure and the cerebrolateral connectives to the anterior body region in early juveniles probably constitute the adult cerebral and frontal nerves but, in contrast to adult specimens, only some of them show weak FMRF-amide-like immunoreactivity (Figure 11O; [60]).

In *Halomenia gravida*, a brooding species that lacks an apical organ, a formation of the nervous system from an anterior and a posterior subsystem was described and it was hypothesized that the latter constitutes the source of the dorsoterminal sense organ [67]. In *Epimenia babai*, development of the longitudinal nerve cords was described as strictly in anterior to posterior direction from the cerebral ganglia [48], which is not the case in *Wirenia argentea* and *Gymnomenia pellucida*, and should be reinvestigated using modern methods.

### Comparative molluscan nervous system development

The pattern of neurotransmitter expression during nervous system development in the Solenogastres shows differences to that of the Polyplacophora, the only other aculiferan taxon for which data are currently available. In both, *Ischnochiton hakodadensis* and *Mopalia muscosa*, the ventral (pedal) nerve cords show FMRF-amide-like immunoreactivity prior to the lateral (pleurovisceral) cords. The same is true for serotonin-like immunoreactivity in *I. hakodadensis*, whereas in *M. muscosa* it appears simultaneously in the ventral and lateral nervous system [43, 44]. In solenogasters, serotonin-like immunoreactivity develops first in the lateral and then in the ventral nervous system (according to the order of formation of the respective structures as is revealed by the acetylated α-tubulin-LIR labeling), and FMRF-amide-like immunoreactivity occurs simultaneously in both systems. Since every neurotransmitter expression pattern reflects the physiological rather than the morphological development (in contrast to the distribution pattern of α-tubulin, which is a structural protein and thus a purely morphological marker), the succession of the appearance of structures in neurotransmitter-labeled specimens does not necessarily reflect the morphological development. This is exemplified by the discrepancies between the development of the serotonin-, FMRF-amide-, and acetylated α-tubulin-like immunoreactivity in solenogasters (this study) or the FMRF-amide- and the serotonin-like immunoreactivity in *M. muscosa* [43]. Thus, it is impossible to decide, in which succession the nerve cords develop in polyplacophorans, since no data on acetylated α-tubulin-like immunoreactivity are available from the respective stages.

Polyplacophora and Solenogastres are similar in possessing at least eight flask-shaped cells in the apical organ, but the distribution pattern of the investigated neurotransmitters is different. The number of serotonin-LIR flask-shaped cells is only two in solenogasters, whereas a number of eight is reported for the two above-mentioned polyplacophoran species [43, 44]. The same holds true for the number of FMRF-amide-LIR flask-shaped cells, which is two in the Solenogastres, while six have been reported in *I. hakodadensis* (FMRF-amide-LIR apical organ cells were also reported for *M. muscosa* but without mentioning their specific form and number). For the serotonin-LIR cells, the timing of development is also different. In polyplacophorans, they appear shortly after hatching and are among the first structures to form, while in solenogasters they were only found in advanced larvae when the trunk had developed considerably and at least the lateral pair of nerve cords had been formed. Acetylated α-tubulin-LIR labeling, however, showed a higher number of flask-shaped cells, thus indicating that the solenogaster apical organ is more complex than revealed by the transmitter labeling. Accordingly, it appears possible that the apical organ cells that were found in polyplacophorans (and entoprocts) [24, 43, 44] are also present in solenogasters and that they simply lack the respective neurotransmitters. Non-flask shaped peripheral cells, which are present in polyplacophoran and some entoproct larvae, were not found in the larvae of the two solenogaster species investigated. Given the morphologically well-founded monophyly of the Tetraneuralia (Mollusca + Entoprocta) [25–27], and the fact that such peripheral cells are also present in the larvae of several gastropods (for example, [68–71]), this is most likely the result of secondary simplification in the Solenogastres – a phenomenon also reflected in their myogenesis [14]. The sensory equipment appears to be generally simpler in the solenogaster larva compared to its polyplacophoran counterpart, since none of the additional serotonin-LIR sensory cells reported for polyplacophorans [43, 44] were found. Furthermore, no indication of an ampullary system was detected, supporting the view that it is an apomorphy of the Polyplacophora [72].

With respect to conchiferan molluscs, a formation of the early larval nervous system from an anterior and a posterior subsystem, as well as an appearance of the lateral nervous system (or at least its scaffolding) before the ventral one were found in gastropods and bivalves based on neurotransmitter labeling [70, 73–75]. Both processes are clearly recognizable in solenogasters with acetylated α-tubulin-LIR labeling, and the earlier formation of the lateral nervous system is also recognizable with serotonin-LIR labeling.

### Shared early and diverging later mechanisms of neural patterning in aculiferan molluscs and polychaetes: nonsegmental nervous system development in molluscs and a basal spiralian pattern

In polychaetes, after more or less synchronous formation of the first three larval segments, the later segments and their related organs form in an anterior to posterior progression from a posterior growth zone, although variations from this scheme are not uncommon [76–82]. During solenogaster late larval development, however, the ventral nervous system, including the clusters of perikarya and the commissures, appears simultaneously. Thus, a teloblastic segment formation pattern as found in annelids is absent in solenogasters and a segmented last common ancestor (LCA) of molluscs is supported by neither our data nor by the numerous previous studies on molluscan - and especially polyplacophoran and solenogaster - neuromuscular development (for example, [14, 43–45]). Interestingly, however, early nervous system development (that is, prior to the establishment of segment formation from the posterior growth zone) in the polychaetes *Phyllodoce maculata*, *Pomatoceros lamarckii,* and especially *Platynereis dumerilii,* shows a particularly striking resemblance to the mode of neural development described here for solenogasters. Based mainly on neurotransmitter labeling in the first two species and on acetylated α-tubulin- and serotonin-LIR labeling in *P. dumerilii,* it was shown that nervous system development likewise starts from the apical and the abapical pole with the apical organ as the anterior neurogenic domain. As in the Solenogastres, the pre- and the post-trochal portion subsequently establish a connection in the region of the prototroch, resulting in one pair of longitudinal neurite bundles. Likewise, a serotonin-LIR ring-like innervation of the prototroch is present [58, 82, 83]. Based mainly on neurotransmitter data, two other polychaete species, *Sabellaria alveolata* and *Spirorbis* sp., however, exhibit a similar morphology but a profoundly different developmental pattern by, for example, lacking a posterior neurogenic domain [81, 84]. Since early development of the nervous system from an anterior and a posterior subsystem also has been described for some polychaetes with direct development [85, 86], it appears possible that not only the anatomy of the larval nervous system, consisting of an apical organ, one pair of longitudinal neurite bundles, and a serotonin-LIR innervation of the prototroch (cf. [87]), but also the specific mode of formation from an anterior and a posterior neurogenic domain, constitutes an ancestral pattern in early nervous system development of spiralians. This scenario is supported by various data on other Spiralia, which show that an anterior (apical) and posterior (abapical) subsystem contributing to the development of the nervous system is also present in larvae of platyhelminths, nemerteans, and probably phoronids [88–91]. In a platyhelminth with direct development, as well as in a brachiopod, a posterior neurogenic subsystem, however, was not found [92, 93].

In almost all spiralians investigated so far (see, for example, the works cited above), a single pair of longitudinal neurite bundles or nerve cords starts to form initially, regardless of the mode of development (direct versus indirect) and the total number of nerve cords eventually present in the adults, which may, for example, range between one and five in annelids [36, 38, 41, 81, 84, 86, 94]. Even some representatives that lack distinct longitudinal nerve cords as adults, such as brachiopods or phoronids, initially form a nervous system including paired longitudinal neurite bundles [91, 93, 95]. Accordingly, we consider this an ancestral feature of spiralians and argue that the LCA of the Spiralia likewise had one pair of longitudinal nerve cords. From this basic pattern modifications, such as tetraneury in Mollusca + Entoprocta or a further increase of longitudinal nerve cords in platyhelminths (the so-called orthogon, cf. [96]) or certain polychaetes, occurred in most subclades. This scenario implies that the orthogon-like multistranded nervous system of the platyhelminths is a derived condition and does not constitute a basal feature of the Spiralia as often proposed (for example, [96]). The Annelida have diverged from all other, nonsegmented spiralian lineages by the establishment of the posterior growth zone and teloblastic growth, involving the formation of neural subsets in a strict anterior-posterior direction.

## Conclusions

Our data on the development of the solenogaster nervous system support a nonsegmented ancestry of molluscs. However, similarities between solenogasters and polychaetes during early nervous system development (that is, prior to the establishment of segment formation from the posterior growth zone in annelids) exist and include the formation of the nervous system from an apical and an abapical neurogenic domain with subsequent fusion of the pre- and post-trochal portion in the region of the prototroch. Thus, the early formation of the larval nervous system and the neural architecture of the early larvae involving an apical organ, two longitudinal neurite bundles, and a serotonin-LIR innervation underlying the prototroch are very similar and both aspects are at least in part shared by other spiralian larvae. We therefore consider these shared neural features as characters of the last common ancestor (LCA) of the Spiralia. After the establishment of the teloblastic segmentation pattern in polychaetes, however, the similarities disappear, since neither solenogasters nor polyplacophorans or any other spiralian representative apart from the annelids shows such a segmental formation of neural structures. This argues for an unsegmented LCA of the Spiralia with segmentation having evolved only along the line leading to the annelids. The either exclusive formation of one pair of longitudinal neurite bundles or its precocious development in comparison to additional bundles during development argues for a spiralian LCA with a single pair of longitudinal nerve cords, implying that deviations from this ground plan, such as tetraneury of the Tetraneuralia or the orthogon of platyhelminths, are derived conditions.

## Abbreviations

ao: apical organ
aoc: flask-shaped cell of apical organ
at: apical tuft
bc: buccal connective
bg: buccal ganglion
block-PTA: 6% solution of normal goat serum in PTA
BOLD: Barcode of Life Data System
c: calymma
cc: cerebral commissure
cg: cerebral ganglion
CLSM: confocal laser scanning microscopy
dph: days posthatching
f: foot
FSSW: 20 μm-filtered and UV-sterilized sea water with a salinity of 35‰
hg: hindgut anlage
ia: innervation of atrial sense organ
ICC: immunocytochemistry
ip: innervation of pedal pit
LCA: last common ancestor
LIR: like immunoreactive
lnb: lateral neurite bundle(s)
lvc: lateroventral connective
mg: midgut anlage
nlpr: neurite to lateral parts of pre-trochal region
np: neuropil of apical organ
npr: neurite to prototroch
pg: pedal ganglion
pnd: posterior neurogenic domain
pnp: prototrochal nerve plexus
pp: pedal pit
pt: prototroch
PTA: 0.1 M phosphate buffer (pH = 7.3) with 4% Triton X-100 and 0.1% sodium azide
RT: room temperature
sna: serotonin-LIR neurites to anterior body region
snt: serotonin-LIR neurites to terminal body region
sp: serotonin-LIR perikaryon
src: suprarectal commissure
TEM: transmission electron microscopy
tr: trunk
tt: telotroch
vnb: ventral neurite bundle
vnp: ventromedian nerve plexus.
